# Pulsed-field ablation for the treatment of left atrial reentry tachycardia

**DOI:** 10.1007/s10840-022-01436-1

**Published:** 2022-12-11

**Authors:** Thomas Kueffer, Jens Seiler, Antonio Madaffari, Aline Mühl, Babken Asatryan, Robin Stettler, Andreas Haeberlin, Fabian Noti, Helge Servatius, Hildegard Tanner, Samuel H. Baldinger, Tobias Reichlin, Laurent Roten

**Affiliations:** 1 Department of Cardiology, Inselspital, Bern University Hospital, University of Bern, Freiburgstrasse, CH-3010 Bern, Switzerland; 2 ARTORG Center, University of Bern, Bern, Switzerland

**Keywords:** Pulsed field ablation, Electroporation, Mitral isthmus, Roof line, Anterior line, Reentry tachycardia, 3D mapping

## Abstract

**Background:**

We describe our initial experience using a multipolar pulsed-field ablation catheter for the treatment of left atrial (LA) reentry tachycardia.

**Methods:**

We included all patients with LA reentry tachycardia treated with PFA at our institution between September 2021 and March 2022. The tachycardia mechanism was identified using 3D electro-anatomical mapping (3D-EAM). Subsequently, a roof line, anterior line, or mitral isthmus line was ablated as appropriate. Roof line ablation was always combined with LA posterior wall (LAPW) ablation. Positioning of the PFA catheter was guided by a 3D-EAM system and by fluoroscopy. Bidirectional block across lines was verified using standard criteria. Additional radiofrequency ablation (RFA) was used to achieve bidirectional block as necessary.

**Results:**

Among 22 patients (median age 70 (59–75) years; 9 females), we identified 27 LA reentry tachycardia: seven roof dependent macro-reentries, one posterior-wall micro-reentry, twelve peri-mitral macro-reentries, and seven anterior-wall micro-reentries. We ablated a total of 20 roof lines, 13 anterior lines, and 6 mitral isthmus lines. Additional RFA was necessary for two anterior lines (15%) and three mitral isthmus lines (50%). Bidirectional block was achieved across all roof lines, 92% of anterior lines, and 83% of mitral isthmus lines. We observed no acute procedural complications.

**Conclusion:**

Ablation of a roof line and of the LAPW is feasible, effective, and safe using this multipolar PFA catheter. However, the catheter is less suited for ablation of the mitral isthmus and the anterior line. A focal pulsed-field ablation catheter may be more effective for ablation of these lines.

**Graphical Abstract:**

This study shows the feasibility to ablate linear lesions with a multipolar pulsed-field ablation catheter. 27 left atrial reentry tachycardia were treated in 22 patients.

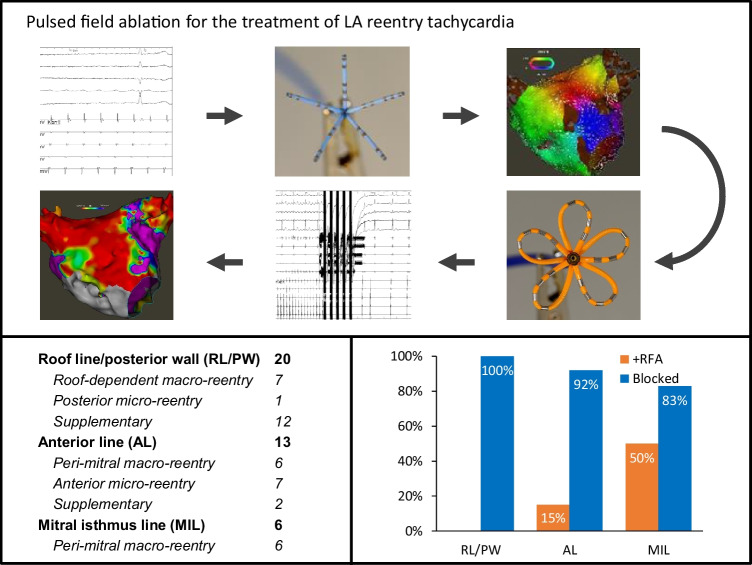

**Supplementary information:**

The online version contains supplementary material available at 10.1007/s10840-022-01436-1.

## Introduction

Reentrant left atrial (LA) tachycardia may develop following LA ablation of atrial fibrillation or occur spontaneously in diseased LA. The main mechanisms of reentrant LA tachycardia are roof-dependent- or mitral isthmus-dependent macro-reentry, or micro-reentry involving scar tissue.

The ablation strategy for LA tachycardia involves ablation of a line across the roof, the mitral isthmus or anterior wall, or focal ablation of scar tissue. However, in 20–30% of LA linear ablations, bidirectional block cannot be achieved acutely. Even if bidirectional block can be achieved, it may recover in up to 40% of cases. Both scenarios can facilitate new arrhythmias [1–3]. Most operators use radiofrequency (RF) energy for LA linear ablations. However, RF energy must be used cautiously, particularly on the left atrial posterior wall (LAPW), to avoid serious side effects such as esophageal damage or perforation. Different strategies have been adopted to overcome the limitations of RF energy. The most successful strategy may be ethanol injection into the vein of Marshal for mitral isthmus line ablation or adjunctive epicardial ablation for roof lines [4–7]. However, these adjunctive approaches are time-consuming and limited to experienced centers.

Pulsed field ablation (PFA) is a novel, non-thermal ablation modality in which cardiac tissue is exposed to repetitive short and intense electrical fields [8]. PFA results in destabilization of cell membranes, formation of pores, and ultimately cell death. Cardiac muscle cells are particularly vulnerable to this effect resulting in selective ablation of the myocardium while sparing other tissues [9]. PFA therefore holds the promise of increased efficacy without sacrificing safety. The first PFA catheter received CE mark in 2021 and is available for clinical use in Europe. The design of this PFA catheter is optimized for pulmonary vein isolation (PVI), but other LA arrhythmias may also be targeted. We report our first experience using a novel pentaspline PFA catheter for LA reentrant tachycardia.

## Methods

### Study population

Consecutive patients with documented LA reentry tachycardia undergoing ablation by PFA at our institution were prospectively enrolled into an institutional registry. The registry was approved by the local ethics committee (approval number PB_2018-00,226), and the study was carried out in accordance with the principles of the Declaration of Helsinki. The authors had full access to and take full responsibility for the integrity of the data.

### Procedural workflow

Our workflow has been described previously [10]. In brief, all patients underwent pre-procedural trans-esophageal echocardiography and cardiac computed tomography to rule out intracardiac thrombi and to obtain a detailed 3D anatomy of the LA. Deep conscious sedation was induced by midazolam, fentanyl, and propofol in an operator-directed, nurse-administered fashion [11]. No paralytics were used. LA access was obtained either by transseptal puncture or through a patent foramen ovale. Heparin was administered to maintain an activated clotting time above 350 s during the procedure. Subsequently, the standard sheath used for LA access was exchanged for a dedicated 13-F steerable sheath (Faradrive, Boston Scientific, Marlborough, MA). Next, a multipolar 3D-mapping catheter (Pentaray, Biosense Webster, Irvine, CA) was introduced through the 13-F steerable sheath. With this catheter, a 3D electroanatomical map (3D-EAM) of the LA was constructed, and the tachycardia mechanism was determined. Reentry mechanisms suitable for ablation by PFA were defined as roof-dependent or peri-mitral macro-reentry tachycardia or scar-related anterior or posterior micro-reentry tachycardia. The mapping catheter was then exchanged for the multipolar PFA catheter, and the pulmonary veins (PVs) were isolated as necessary [12]. Additional ablations were applied to create appropriate linear lesions to target the reentrant tachycardia. In the case of roof-dependent reentry, we performed a roof line, followed by complete LAPW isolation. For anterior scar-related reentry or peri-mitral macro-reentry with an extensive anterior scar, an anterior line was ablated by placing lesions between a superior pulmonary vein and the high anterior mitral annulus. For peri-mitral macro-reentry without an extensive anterior scar, we ablated a standard mitral isthmus line from the left inferior pulmonary vein (LIPV) to the posterior mitral annulus. PVI was confirmed by testing for bidirectional block.

### Ablation system

The PFA platform, called the FARAPULSE PFA System (Boston Scientific, Marlborough, Massachusetts), includes a generator (Farastar), which produces the therapeutic electrical field, a 12F over-the-wire multipolar ablation catheter (Farawave), available in two sizes (31 mm and 35 mm), and a 13-F steerable sheath (Faradrive). The platform has been described in detail previously and is CE Marked with an indication for PVI in patients with paroxysmal atrial fibrillation [8]. The multipolar ablation catheter has five splines containing four electrodes each. Intracardiac electrograms can be recorded in between splines from the third-to-distal electrode of each spline. The five splines join at their proximal and distal end and can take any intermediary form between a spherical “basket” shape to a fully-deployed “flower” configuration. For ablation, microsecond-scale, high-amplitude electrical pulses are applied between all 20 electrodes in a bipolar, biphasic fashion. This creates a complex electrical field around the catheter and results in irreversible electroporation of any susceptible cells within this field. We chose a peak voltage of 2.0 kV for all ablations and used the 31 mm or 35 mm device according to operator choice.

### Visualization of the PFA catheter on the 3D-EAM system

**Fig. 1:**
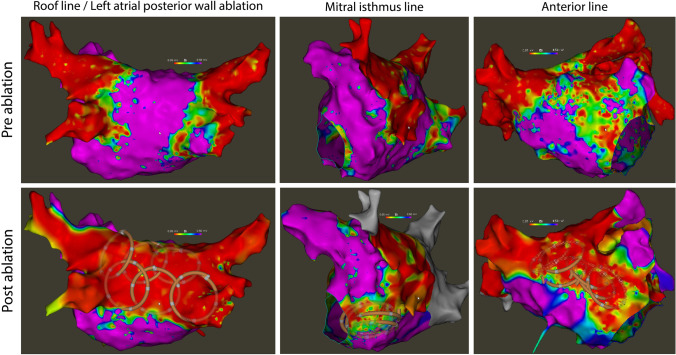
Pre- and post-ablation maps for left atrial linear ablations using a multipolar PFA catheter. Left atrial 3D-electroanatomical bipolar voltage maps for posterior wall isolation, mitral isthmus line ablation, and anterior line ablation using a multipolar PFA catheter. Pre-ablation maps were acquired during left atrial reentry tachycardia, post-ablation maps during sinus rhythm. The PFA catheter can be tracked by the 3D-EAM system and its position visualized in a simplified circular shape (lower row). Flower and basket shape cannot be distinguished. The scale for bipolar voltage maps is 0.05 mV to 0.5 mV. PFA = pulsed field ablation

**Fig. 2:**
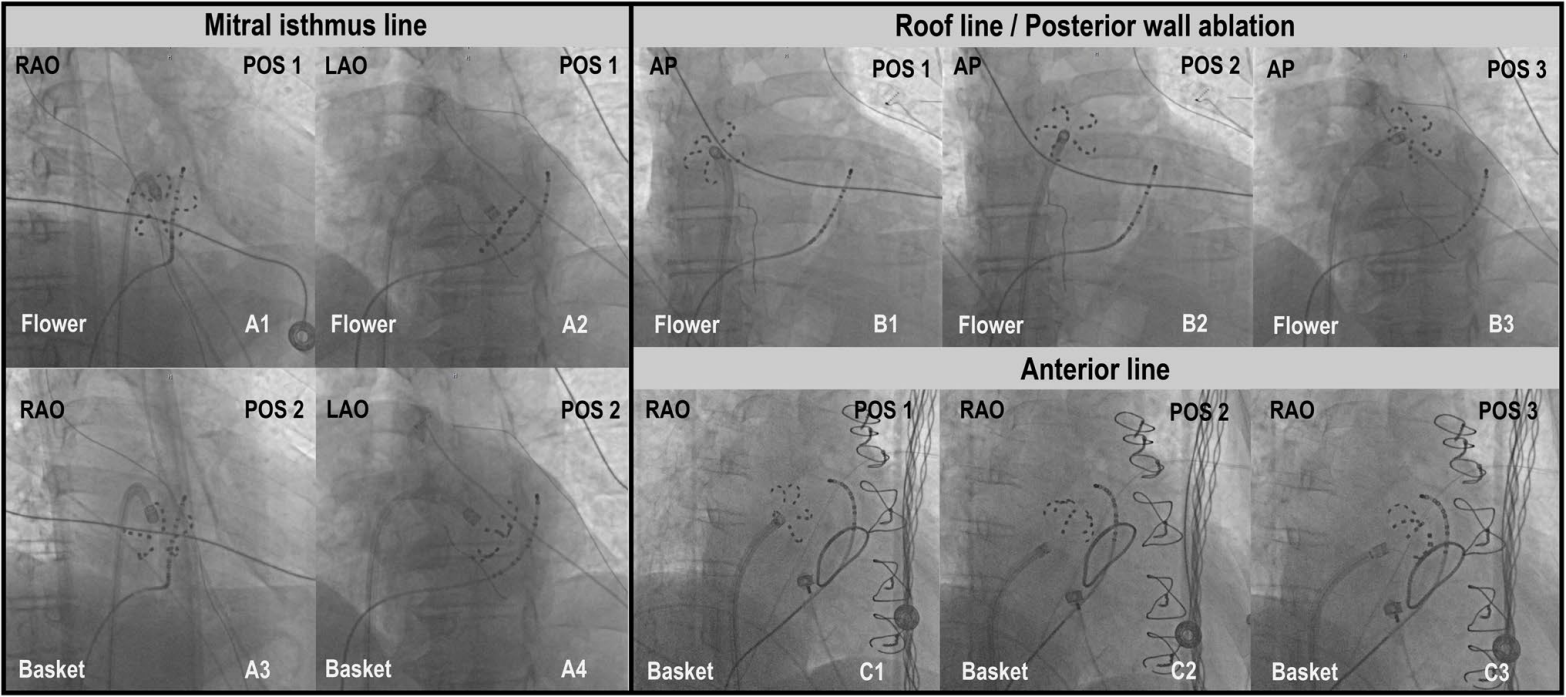
Fluoroscopic guidance of catheter positioning during left atrial linear ablations using a multipolar PFA catheter. **A1**–**A4** Ablation of a mitral isthmus line with the PFA catheter in either flower (**A1** und **A2**) or basket configuration (**A3** und **A4**). **B1–B3** Roof line ablation with the catheter in flower configuration in different positions on the upper posterior wall. **C1-C3** Catheter positioning for anterior line ablation. The flower configuration was used for the superior part of the anterior line (**C1**) and the basket configuration for ablation closer to the mitral annulus (**C2** and **C3**). Lesion overlap was further verified by a 3D-EAM system (not shown here). PFA = pulsed field ablation; 3D-EAM = 3D-electroanatomical mapping

For 3D-EAM, we used both the Carto (Carto 3, Biosense Webster, Irvine, CA) and Rhythmia system (Boston Scientific, Marlborough, MA). Visualization of the PFA catheter in the Carto system was realized by defining a custom 6F circular catheter with six 2-mm electrodes and 17-mm center-to-center inter-electrode spacing. Since the catheter only has five splines, we split the signal of the first electrode and routed it to the additional sixth pin, consequently displaying the curved shape always as a closed loop. Catheter position was verified on 3D-EAM and fluoroscopy before ablation (Figs. 1 and 2). The location pad of the 3D-mapping system was disabled during ablation to speed up visualization recovery thereafter. Visualization of the PFA catheter in the Rhythmia system was done as reported elsewhere [13].

### Roof line

With the guidewire in the superior PVs, an anchoring ablation was first performed with the catheter in flower configuration and the steerable sheath rotated towards the posterior wall. The remaining distance between the anchoring lesions in the LSPV and the RSPV was covered with overlapping ablations in flower configuration and with the guidewire retracted inside the catheter (Fig. 2). One ablation consisted of four applications in flower configuration, rotating the device 30°–40° after the first pair of applications for circumferential coverage. Another set of ablations in the same fashion on a more inferior aspect of the posterior wall was applied to complete LAPW isolation (Fig. 1). Complete LAPW isolation was confirmed by 3D-EAM showing no remaining signals on the posterior wall. In the case of remaining signals, PFA was repeated until LAPW isolation was complete.

### Mitral isthmus line

The mitral isthmus line was ablated with the PFA catheter in flower or basket configuration, whichever yielded the best contact and coverage of the area of interest. Lesions were applied from the LIPV towards the mitral annulus (Figs. 1 and 2). If a bi-directional block according to standard criteria could not be achieved by PFA applications alone, the catheter was exchanged, and additional RF ablation was performed endocardially using 35 W, and epicardially in the great cardiac vein, using 20 W.

### Anterior line

An anterior line was ablated connecting the RSPV to the anterior mitral annulus. An anchoring ablation was performed in the RSPV, with the device in flower configuration and the wire in the RSPV, rotating the sheath anteriorly. The same flower configuration was maintained for further applications on the superior aspect of the anterior wall, after retraction of the wire. For locations close to the anterior mitral annulus, ablations were performed with the catheter in basket configuration, ensuring tissue contact of one or two splines in the 3D-EAM (Figs. 1 and 2). For each ablation site, four applications were performed, rotating the catheter by 30°–40° after the first pair of applications. Bi-directional block across the anterior line according to standard criteria was assessed after completion of lesions. Additional PF or RF ablations were performed if no bi-directional block was achieved.

### Statistical analysis

Continuous variables are presented as mean ± standard deviation or as median and interquartile range as appropriate. For statistical analyses, we used R 4.0.2 (R Core Team, Vienna, Austria).

## Results

### Patient characteristics

Patient and procedural characteristics are summarized in Table 1. The cohort consisted of 22 patients (59% male) with a median age of 70 years (IQR 59–75). Seventeen patients (77%) had prior LA ablation procedures, which included PVI in all and previous linear ablations in six cases (27%).

**Table 1:** Patient and procedural characteristics

	*N* = 22
*Patient characteristics*	
Age, years	70 (59–75)
Male sex	13 (59%)
BMI, kg/m^**2**^	26 (26–28)
Prior LA ablation procedures	17 (77%)
Number of prior LA ablation procedures	2 (1–2)
Prior LA linear ablation	6 (27%)
Left atrial volume index, ml/m^2^	47 (43–54)
LVEF, %	55 (45–60)
Congestive heart failure	3 (14%)
Hypertension	11 (50%)
Diabetes	3 (14%)
Stroke or TIA	3 (14%)
CHA_2_DS_2_VASc score	3 (2–3)
0	2
1	3
2	6
3	6
4	4
> 4	1
*Procedural characteristics*	
Procedure time, min	177 (138–202)
Total fluoroscopy time, min	29 (19–37)
Total fluoroscopy dose, cGy*cm^2^	747 (283–1389)

Values are presented as median (interquartile range), or numbers (%). BMI = body mass index; CAD = coronary artery disease; LA = left atrium; LVEF = left ventricular ejection fraction; TIA = transient ischemic attack

An overview of the mapping findings and the ablation lesion sets is presented in Tables 2 and 3. We used the 31-mm PFA device in 19 cases and the 35-mm PFA device in the remaining three cases. In twelve patients, all pulmonary veins were already isolated, and in five patients, the pulmonary veins were re-isolated by PFA. The remaining five patients had their first PVI by PFA. A total of 27 LA reentry tachycardia were observed and targeted by PFA. This included seven roof-dependent macro-reentries, 12 peri-mitral macro-reentries, one micro-reentry involving the posterior wall, and seven micro-reentry involving the anterior wall. We observed no acute procedural complications.

**Table 2:** Overview of the mapping findings and the ablation lesion sets for each patient

Case #	Age, y	Sex	Previous ablations	Arrhythmias present	PFA targets	RFA targets	Blocked lines
1	51	M	PVI	Roof-dependent, peri-mitral^†^	PWI, AL, PVI	-	RL, AL
2	69	M	PVI	Roof-dependent^†^, peri-mitral^†^	PWI, AL	-	RL, AL
3	38	M	PVI	Anterior reentry^†^	PWI, AL^‡^	-	RL
4	70	F	PVI	Anterior reentry^†^	PWI, AL	-	RL, AL
5	59	M	PVI, SVC, MIL	Peri-mitral^†^	PWI, MIL	-	RL, MIL
6	72	M	PVI	Roof-dependent^†^, peri-mitral	PWI, MIL, PVI	-	RL, MIL
7	62	F	PVI	Peri-mitral^†^	PWI, MIL, PVI	MIL	RL, MIL
8	75	M	PVI, MIL	Roof-dependent^†^	PWI	-	RL, MIL
9	75	F	PVI, SVC	Anterior reentry^†^, peri-mitral	PWI, AL	AL	RL, AL
10	43	M	PVI	Peri-mitral^†^	PWI, MIL	MIL	RL, MIL
11	75	F	-	Anterior reentry^†^	AL, PVI	AL	AL
12	65	M	-	Peri-mitral^†^	PWI, AL, PVI	-	RL, AL
13	69	F	PVI, AL	Roof-dependent	PWI, AL	-	RL, AL
14	71	F	PVI	Anterior reentry, peri-mitral^†^	PWI, AL	-	RL, AL
15	76	M	PVI	Anterior reentry^†^	AL, PVI	-	AL
16	71	F	PVI	Anterior reentry^†^	PWI, AL	-	RL, AL
17	82	M	-	Peri-mitral^†^	PWI, MIL^‡^, PVI	-	RL
18	75	M	-	Reentry within posterior wall†	PWI, PVI	-	RL
19	81	F	PVI, AL, Focal	Roof-dependent	PWI, AL	-	RL, AL
20	58	F	-	Roof-dependent^†^	PWI, PVI	-	RL
21	71	M	PVI, RL, SVC	Peri-mitral^†^	PWI, AL	-	RL, AL
22	57	M	PVI, RL	Peri-mitral	PWI, MIL	MIL	RL, MIL

^†^Terminated with PFA application. ^‡^Line not blocked. AL, anterior line; MIL, mitral isthmus line; PFA, pulsed field ablation; PVI, pulmonary vein isolation; PWI, posterior wall isolation; RFA, radiofrequency ablation; RL, roof line; SVC, superior vena cava

**Table 3:** Overview of ablation lesion sets, targeted arrhythmias and acute outcomes according to anatomical sites

	*N*	Touch up RF	Bi-directional block
Roof line and posterior wall	**20**	**0**	**20 (100%)**
Ablation of RL/PW for arrhythmia	8	-	8 (100%)
Roof-dependent macro-reentry	7	-	7 (100%)
Micro-reentry of posterior wall	1	-	1 (100%)
Supplementary ablation of RL/PW	12	-	12 (100%)
Anterior line	**13**	**2 (15%)**	**12 (92%)**
Ablation of anterior line for arrhythmia	11	2 (18%)	10 (91%)
Peri-mitral macro-reentry	6^†^	1 (17%)	6 (100%)
Anterior wall micro-reentry	7^†^	2 (29%)	6 (86%)
Supplementary ablation of anterior line	2	-	2 (100%)
Mitral isthmus line	**6**	**3 (50%)**	**5 (83%)**
Ablation of mitral isthmus line for arrhythmia	6	3 (50%)	5 (83%)
Peri-mitral macro-reentry	6	3 (50%)	5 (83%)
Supplementary ablation of mitral isthmus line	-	-	-

^†^Two patients had both peri-mitral macro-reentry as well as anterior wall micro-reentry. PW = posterior wall; RL = roof line

### Roof-dependent macro-reentry and posterior, scar-related micro-reentry

Of the seven roof-dependent macro-reentries and one micro-reentry involving the posterior wall, five (63%) terminated during ablation of the roof line. For all patients receiving roof line ablation, LAPW isolation was also performed. Furthermore, a supplementary roof line and LAPW isolation was applied in 12 patients to avoid future occurrence of roof-dependent arrhythmia (Tables 2 and 3). No additional RF ablation was needed in any of the 20 patients to complete the roof line or to isolate the LAPW. No atrio-esophageal fistula occurred.

### Peri-mitral macro-reentry

Among the twelve patients with ongoing peri-mitral macro-reentry, a standard mitral isthmus line was applied in six patients (50%) and an anterior line in the remaining six (50%; Tables 2 and 3). The latter all had an extensive anterior scar (Supplemental Fig. 1), and two showed anterior micro-reentry as well. Peri-mitral tachycardia terminated with PFA of the standard mitral isthmus line in four (67%) patients and during ablation of the anterior line in 6 (100%). Additional RF ablation to achieve bidirectional block of the mitral isthmus line and anterior line was needed in three (50%) and two (40%) patients respectively (35 W for endocardial ablation, 20 W for epicardial ablation in the great cardiac vein). In patients requiring additional RF ablation for the completion of the mitral isthmus line, RF ablation was performed within the great cardiac vein for all three cases and endocardially in one case. Bidirectional block of the standard mitral isthmus line and the anterior line was finally and acutely achieved in five (83%) and six (100%) patients with peri-mitral macro-reentry tachycardia, respectively.

In one patient, PFA of the anterior line resulted in transient, complete AV block, which led us to switch to RF ablation. However, transient, complete AV block also occurred with RF ablation and was finally attributed to a vagal reaction during ablation. After the application of 0.5 mg atropine, RF ablation at the same site was possible without the occurrence of complete AV block.

### Anterior, scar-related micro-reentry

**Fig. 3:**
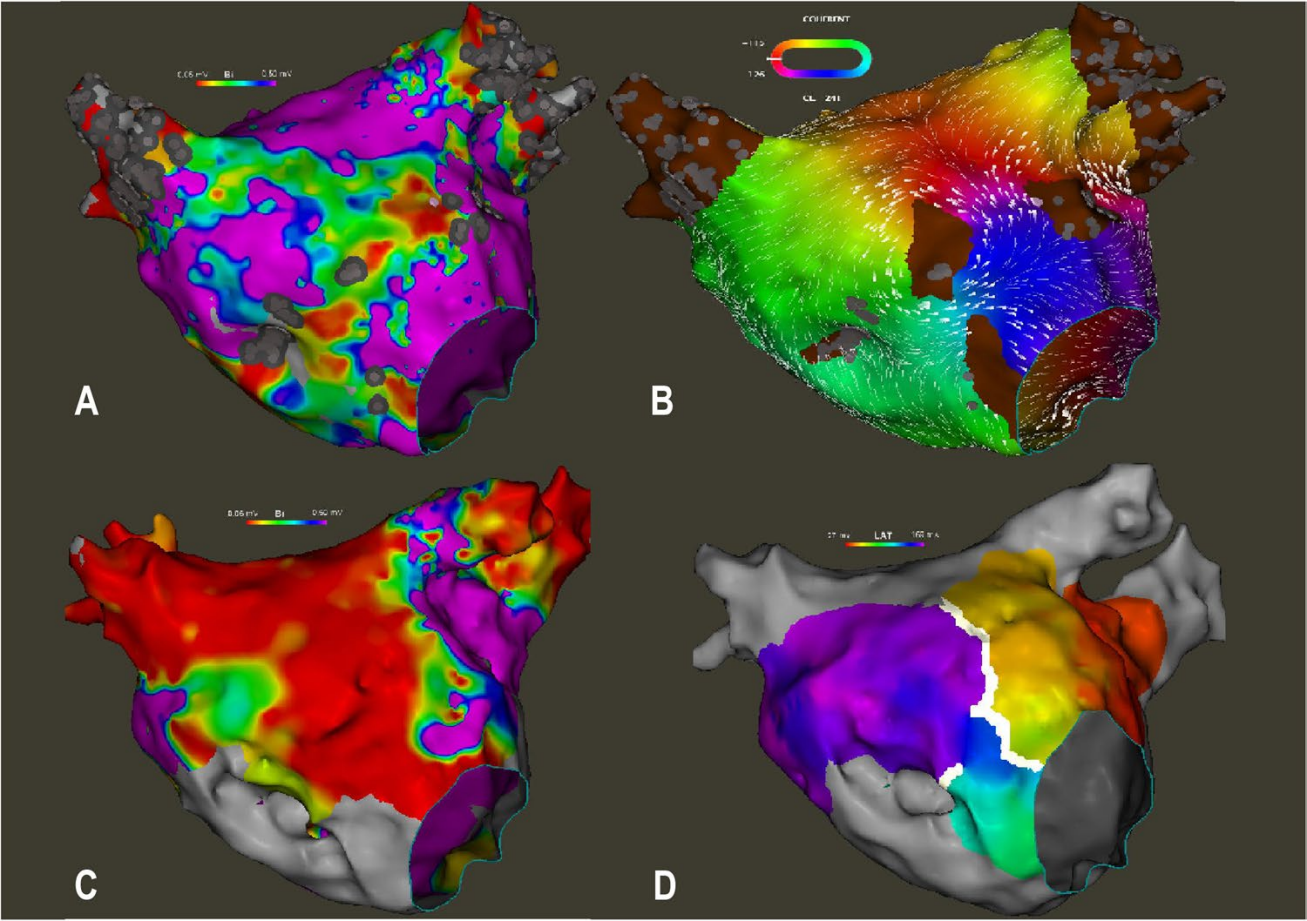
Anterior scar related reentry tachycardia. Bipolar voltage map (**A**) and local activation map (**B**) of a representative scar-related reentry tachycardia on the anterior wall of the left atrium. After ablation of an anterior line, the voltage map (**C**) displays a large, homogenous line of low amplitude. Activation map during pacing from the left lateral wall shows a blocked anterior line (**D**, white line). The scale for bipolar voltage maps is 0.05 mV to 0.5 mV

Of the seven anterior micro-reentry tachycardias, 6 (86%) terminated during PFA of the area of origin within an anterior scar region (Fig. 3). Two patients with anterior micro-reentries also had peri-mitral macro-reentry (see above). In five patients, the anterior line was applied using PFA exclusively. In the remaining two patients, additional RF ablation was necessary to achieve bidirectional block. One of those had an LAA occluder (Amplatzer Amulet, 31 mm), which prevented the delivery of PFA energy due to electrical interference with the catheter. Bidirectional block across the anterior line was not achieved in one patient (14%).

## Discussion

In this cohort of 22 patients, we report our initial experience using a commercially available PFA catheter for the treatment of LA reentry tachycardia. Of the 39 lines attempted, a roof line and LAPW isolation was the target in 20 patients, an anterior line in 13, and a standard mitral isthmus line in 6. Bidirectional block could be achieved across 37 (95%) of the targeted lines, with supplementary RF ablation in 5 (14%) cases.

Due to the combination of procedural efficacy, procedural safety, and lesion durability, the non-thermal PFA technology has the potential to become the preferred technology for atrial fibrillation ablation. Most of the PFA work to date has focused on PVI [8, 12, 14, 15]. Accordingly, the design of the currently available PFA catheter has been optimized for PVI [8, 10, 14]. The advantages of PFA however make the technology also attractive for use outside the PVs [13, 16]. The use of the currently approved PFA catheter for linear ablation in patients with LA reentry tachycardia has been reported in case reports but was not systematically studied [17].

### Roof line and left atrial posterior wall isolation

The multipolar PFA catheter is particularly well suited for the application of a roof line and for LAPW isolation. In comparison, ablation of a roof line or LAPW isolation by RF ablation is challenging. In a meta-analysis, the success rate for LAPW isolation with RF ablation was 71% (95% CI, 46–92%) [18]. Pak et al. used 3D-EAM guided touch-up ablations on local signals within the posterior wall in addition to a roof and posterior inferior line and reached a high success rate of acute, complete LAPW isolation in 94% of cases [19]. Those extended RF ablation strategies are comparable with LAPW isolation using PFA in terms of ablation area. However, PFA might have some distinct advantages over RF ablation: First, with RF, extensive ablation of the posterior wall must be applied cautiously to avoid esophageal damage. In that regard, the tissue-selective properties of PFA should reduce the risk of esophageal damage even with large-area ablation [20–23]. Indeed, two studies found no esophageal lesions on post-procedural esophagogastro-duodenoscopy after LAPW isolation using the same PFA system [13, 14]. Second, Pambrun et al. showed the importance of epicardial bypass in cases with residual gaps in the roof line [5]. By covering the entire posterior wall, the PFA approach also covers potential epicardial insertions into the LAPW.

In a pre-market trial, the same PFA device that was used in our study was successful in LAPW isolation in 25/25 patients [13, 14]. With a different PFA catheter platform allowing for focal lesions, application of a roof line using PFA was effective in 22/22 patients [24]. In aggregation, this means that the lesion depth of PFA at the roof and the posterior wall seems sufficient to also target epicardial bundles.

### Standard mitral isthmus line

Ablation of a standard mitral isthmus line with RF ablation is successful in achieving bidirectional block in only 64% of patients [1]. The mitral isthmus is crossed by both the left circumflex coronary artery and the great coronary vein on the mitral side and by the vein of Marshal on the pulmonary venous side. These vessels are surrounded by myofibres, providing an epicardial, electrical bypass across linear lesions that are applied on the endocardial side [1]. Furthermore, the cooling effect of the blood flow of these vessels protects the tissue surrounding the veins from heating by RF ablation. To overcome these limitations, ethanol ablation of the vein of Marshal has proven very effective, although additional RF ablation on the mitral side of the mitral isthmus line or within the great cardiac vein is still necessary for a majority of patients [4, 7].

Using PFA, we were able to achieve acute bidirectional block across the mitral isthmus line in 83% of cases, including three cases in which additional RF ablation within the great cardiac vein was needed. Since the successful ablation of standard mitral isthmus line might still request additional ablation from within the great cardiac vein, a focal PFA catheter may be better suited. In a pre-clinical study, a different PFA system was used to deliver a combination of pulsed field and RF lesions from a single lattice-tip catheter both endo- and epicardially and achieved acute mitral isthmus block in 13/14 patients [24].

It remains to be demonstrated, whether the necessary lesion depth for durable block of the standard mitral isthmus line can be achieved by PFA. Gunawardene et al. reported successful mitral isthmus ablation using PFA in two patients without the need for additional RFA. However, in one case, coronary artery spasm occurred after eight PFA applications and resolved upon administration of nitroglycerin [17]. Although coronary arteries did not display particular susceptibility to PFA in animal models[23], more recent evidence showed that PFA routinely provokes coronary spasms when energy is delivered adjacent to coronary arteries [25]. In view of this new data, we currently avoid PFA in close proximity to large coronary arteries.

In our study, for the ablation of the mitral isthmus line, PFA lesions were applied in both the flower and basket configuration. The flower configuration can be used to target a larger area. The basket configuration might be used to optimize tissue contact of some splines of the PFA catheter at sites with residual gaps identified by 3D-EAM subsequent to PFA applications in the flower configuration.

### Anterior line

Increased tissue thickness and epicardial connections may be obstacles to the application of an anterior line from the RSPV to the anterior mitral annulus. Ablation of an anterior line usually is not the first choice for the treatment of peri-mitral macro-reentry. However, this line might be the preferred approach in the presence of an extensive scar on the anterior wall, forming the substrate for both peri-mitral macro-reentry and anterior micro-reentry tachycardia. Using RF ablation, a report on a large cohort of 398 patients observed an acute success rate for block across an anterior line of 64% [3]. In our experience, the specific, PV-focused over-the-wire design of the multipolar PFA catheter seems suboptimal for ablation of an anterior line. The superior part of the line close to the RSPV was best attainable with the catheter in the flower configuration. A basket configuration was better suited to reach the mitral side of the anterior line. In particular, it is difficult to maintain good tissue contact on the anterior line, which is also a prerequisite for successful lesion application [26]. Despite extensive PFA, additional RF lesions were necessary in 2 cases (15%) to obtain bidirectional block and one anterior line could not be blocked, resulting in an acute success rate of 92%.

### Integration of the PFA catheter into 3D-EAM systems

The multipolar PFA catheter can be used with simple fluoroscopic guidance and without the need for a 3D-EAM system. However, for planning and delivery of more complex lesion sets than PVI, real-time visualization using a 3D-EAM system allows for verification of appropriate catheter position before ablation. To identify the tachycardia mechanism of LA reentry tachycardia, 3D-EAM is used either way, and integration of the PFA catheter is an obvious option. Despite the current lack of magnetic tracking, impedance-based visualization of the multipolar PFA catheter in two established mapping systems was remarkably accurate. Overlap between lesions as well as proximity to the LA tissue can easily be verified and the catheter position adjusted as needed (Fig. 1). In addition, the identification of electrodes and their corresponding signals may help to identify residual gaps.

### General remarks

Pulsed-field ablation can result in local myocardial capture, thereby terminating the tachycardia by the mechanism of overdrive pacing. Therefore, termination by PFA should not be interpreted as proof of the tachycardia’s mechanism nor as proof for an effective lesion application. Extensive use of PFA for linear ablation and LAPW isolation may impair left atrial pump function. Nakatani et al. compared the effect of PFA with radiofrequency ablation on left atrial mechanical and structural characteristics after pulmonary vein isolation [27]. They found better left atrial reservoir and pump function late after PFA compared to radiofrequency ablation. However, whether this also holds true for PFA outside of the pulmonary veins will need to be addressed by future studies.

### Limitations

Potential limitations of the presented study merit consideration. First, this is a small cohort and represents a single-center experience at an early stage of the implementation of a new technology. Therefore, conclusions on the efficacy and safety of linear PFA should be drawn cautiously. Our results need external validation, ideally by the contribution of multiple centers. Second, the PFA system utilized in this study is labeled and designed for PVI. Dedicated PFA catheters for linear ablation have been developed, are undergoing pre-clinical testing, and will further improve results. Third, we report the acute results to achieve block across ablation lines. Despite favorable evidence accumulating on the durability of PFA lesions in PVI, the durability of linear PFA lesions is currently not known. Fourth, our experience is limited to a specific PFA system. Our results cannot be applied to other PFA platforms as they incorporate different ablation parameters and catheter designs.

## Conclusion

Ablation of a roof line and LAPW isolation is feasible, effective, and safe with this multipolar PFA catheter. However, the catheter is less suited for ablation of the mitral isthmus and the anterior line, for which complementary RF ablation may be needed to achieve bidirectional block. A focal pulsed-field ablation catheter may be more effective for ablation of these lines.

## Supplementary information

Below is the link to the electronic supplementary material.

Supplementary file1 (JPG 4582 KB)

## Data Availability

The authors had full access to and take full responsibility for the integrity of the data.
