# Supplementary figures and images for: Pulsed-field ablation for the treatment of left atrial reentry tachycardia
Source: J Interv Card Electrophysiol. 2022 Dec 11;66(6):1431–40. doi: 10.1007/s10840-022-01436-1 (PMC10457215; doi:10.1007/s10840-022-01436-1)

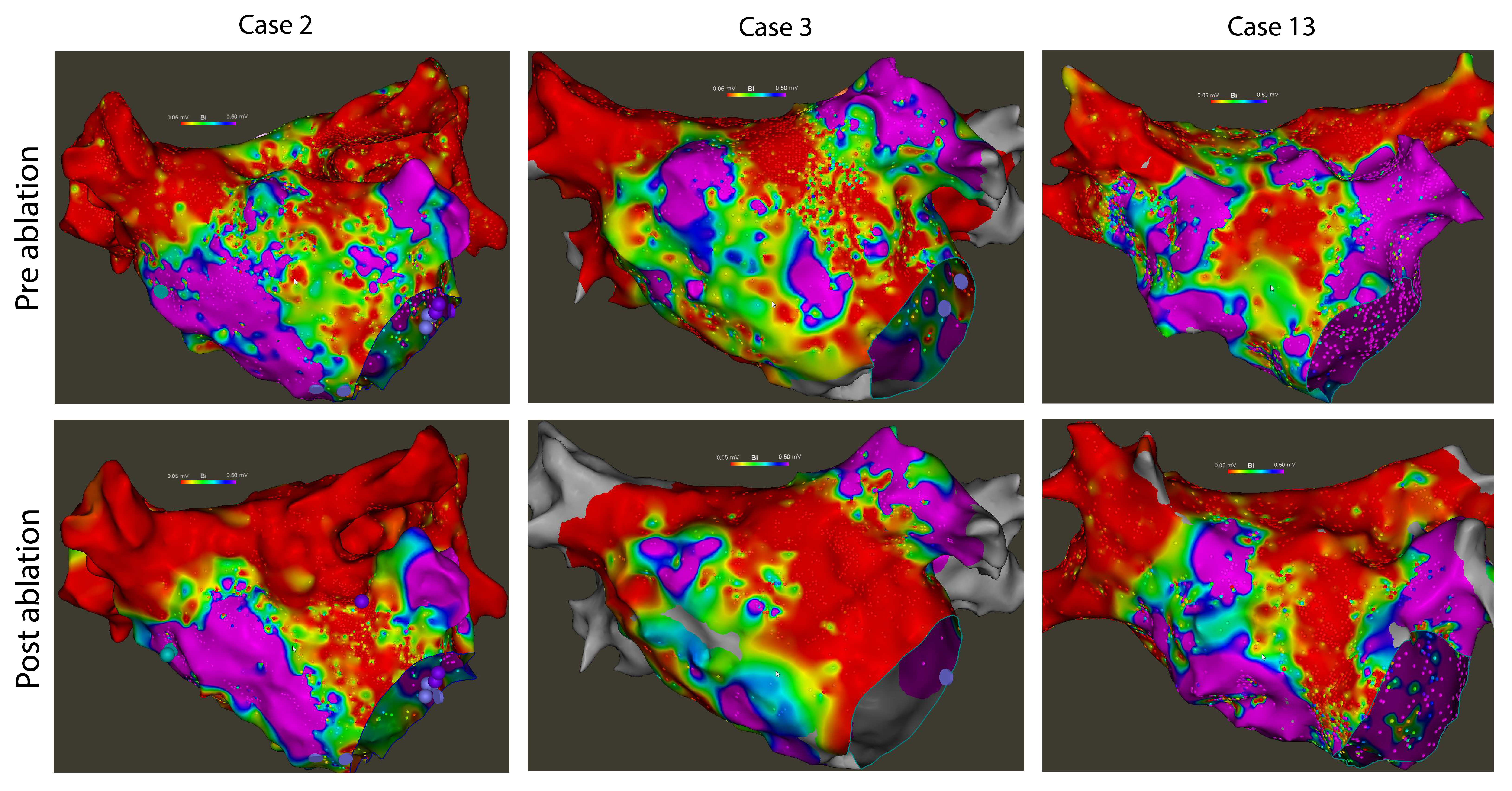

Supplement: Supplementary file 1 — Supplementary file1 (JPG 4582 KB) [file 10840_2022_1436_MOESM1_ESM.jpg]
